# Evaluation of the Completeness and Timeliness of National Malaria Surveillance System in Qatar, 2016

**DOI:** 10.7759/cureus.2851

**Published:** 2018-06-21

**Authors:** Mohamad A Chehab, Mohamed O Bala, Ayman Al-Dahshan, Nagah A Selim, Hamad E Al-Romaihi, Mohammed Al-Thani, Elmoubasher A Farag

**Affiliations:** 1 Community Medicine Residency Program, Hamad Medical Corporation, Doha, QAT; 2 Department of Public Health and Preventive Medicine, Cairo University School of Medicine, Cairo, EGY; 3 Ministry of Public Health, State of Qatar, Doha, QAT; 4 Health Protection and Communicable Disease Control, Ministry of Public Health, Doha, QAT

**Keywords:** malaria, surveillance system, qatar, evaluation

## Abstract

Background

The high influx of migrant workers from malaria-endemic countries along with the presence of a malaria vector in Qatar has raised the alarm of the possible reintroduction of local transmission. Meanwhile, the Qatar Malaria Surveillance System aims to detect any local malaria transmission as well as to monitor trends in imported cases.

Aim

Evaluating the attributes of the Malaria Surveillance System in Qatar will help identify any gaps necessitating rectification.

Method

The completeness and timeliness of the malaria surveillance system were determined. The direct method was used to determine completeness. Timeliness was evaluated by calculating the time lag between the onset of disease and notification receipt by the surveillance team (T) or diagnosis (T1) and between the diagnosis and receipt of notification by the surveillance team (T2).

Results

The overall external completeness of Malaria surveillance system was yielded at 47% (219/493). The most frequently reported data fields were found to be age, gender, and nationality with a percentage of 99% or more. However, the least reported data components were found to be lab results, types of samples, sample collection, and travel destinations with percentages of 59%, 58%, 56%, and 41%, respectively.The overall median time lags was six days for T, four days for T1, and two days for T2.

Conclusion

Our study has identified several merits and areas of improvement in the National Malaria Surveillance System in Qatar. The attributes of evaluation, completeness and timeliness, need more quality improvement. Evaluation of other surveillance system attributes is highly recommended.

## Introduction

Malaria is a vector-borne parasitic disease transmitted through the bite of the female Anopheles mosquito. According to the World Health Organization (WHO) fact sheet on malaria (updated December 2016), 92 countries were affected by malaria in 2015, where Sub-Saharan Africa had the lion’s share of the global disease burden. Additionally, malaria accounted for 212 million cases and 429,000 deaths during 2015. As a result of effective international efforts to prevent and control malaria, the incidence and mortality rates dropped by 37% and 60%, respectively, over the period between 2000 and 2015 [1]. However, fears remain about the emergence and reemergence of malaria as a result of multiple factors, such as global warming, loss of biodiversity due to habitat alteration [2], and invasion of new geographic areas by synanthropic species [3].

The World Health Organization defines malaria elimination as an “interruption of local transmission (reduction to zero incidence of indigenous cases) of a specified malaria parasite species in a defined geographical area as a result of deliberate activities” [4]. In 2015, there were about 3,800,000 cases of malaria and an associated 7,300 deaths in the Eastern Mediterranean region; each accounting for 2% of the respective global figures [5]. Interestingly, according to a WHO analysis, Saudi Arabia and Iran are the two countries in the aforementioned region with the potential to eliminate local malaria by 2020 [6]. However, only three countries in the region (Oman, Syria, and Iraq) have successfully interrupted the local transmission of malaria while two were certified as malaria-free (United Arab Emirates and Morocco) between 2007 and 2015 [7].

In 2012, the state of Qatar was added by the WHO Malaria Elimination Programme to the supplementary list of countries where malaria never existed or disappeared without specific measures [8]. Moreover, Qatar has successfully eliminated the local transmission of malaria in 1970 [9]; however, imported cases of malaria are still reported in the country as a result of the influx of adult male migrants from malaria-endemic countries [10]. Thus, Qatar is currently in the stage of the prevention of the reintroduction of the local transmission of Malaria.

Nevertheless, several studies on the vector of malaria in Qatar raised alarming results. Moreover, a mosquito survey conducted at the Al Rayyan municipality, located in the western region of Qatar, between 2014 and 2015 has revealed the presence of Anopheles stephensi (a malaria vector). Thus, it was concluded that the combined presence of breeding sites and malaria-infected individuals elevate the risk of malaria reintroduction to Qatar [11]. These findings have been supported by other researches conducted in the northeastern region of Al Khor, which also revealed the presence of the established malaria vector Anopheles stephensi [12-13]. Another mosquito larval survey conducted in six sites across Qatar (Alwakra, Alkaraana, Rawdat Alfaras, Hazm Almurkhiya, Nuaija, Alkhor), between 2013 and 2015, has identified that the Anopheles genera constituted 2.6 % of 3,085 collected mosquito larvae [14]. These findings raise alarm about the importance of implementing a robust malaria surveillance and control program in Qatar.

In Qatar, the malaria surveillance system is passive surveillance, which relies on the routine notification of cases by treating physicians in healthcare facilities. There is a need to evaluate the current national malaria surveillance program in the country in order to identify any potential gaps for mitigation and ultimately improving its utility and efficiency while also advocating the importance of strengthening such programs in concordance with the establishment of a dedicated vector surveillance program. The evaluation of any national health surveillance programs could encompass the assessment of several surveillance attributes, such as the simplicity, flexibility, acceptability, completeness, sensitivity, timeliness, representativeness, and stability. Up to the best of our knowledge, no previous study has attempted to evaluate an infectious disease surveillance program at a national level in Qatar, except for one conducted by Nazzal et al. in 2008, which evaluated the national measles surveillance program [15]. Thus, we aimed to evaluate the timeliness and completeness of the national malaria surveillance system in Qatar, which are the most frequently considered attributes of data reporting quality in any surveillance system evaluation.

## Materials and methods

Setting and sampling

Qatar’s national surveillance system categorizes notifiable diseases into two classes depending on national and international regulations of disease reporting. Diseases that are immediately notifiable (within 24 hrs) through telephone or fax are denoted as Class A while those that may be notified as soon as possible (up to one week) are designated as Class B. Standard definitions for case reporting are currently being developed along with guidelines and standard operating procedures for disease surveillance, reporting, and control. As of 2018, the list of notifiable diseases in the State of Qatar encompasses 67 diseases, including malaria, which is classified as a Class B. The surveillance system relies mainly on indicator-based surveillance in which data is collected and input manually due to the absence of an electronic system.

A retrospective records review was conducted at the surveillance unit in the Ministry of Public Health (MoPH), Qatar, to analyze all malaria notifications in the year 2016 for the completeness and the timeliness of notifications. The principal investigators conducted data collection and analysis between March and April 2017. A total of 236 notification paper forms for suspected malaria cases were analyzed.

The malaria reporting sites were classified into six categories: Hamad Medical Corporation (HMC) facilities which include; Hamad General Hospital (HGH), Al Khor Hospital (AKH), Al Wakra Hospital (AWH), and The Cuban Hospital (CH), Primary Health Care Corporation (PHCC) health centers, Qatar Red Crescent (QRC), Qatar Petroleum (QP), the private sector, and others. HMC is the primary governmental provider of hospital care for the residents in Qatar. PHCC is a governmental provider of primary health care (and some secondary health care) through 23 health care centers dispersed across Qatar. QP is the main oil company in Qatar and provides its workers and their families with primary as well as secondary health care services. QRC is responsible for primary health care of single male workers through its health centers.

Data collection

The first attribute of the evaluation, completeness, was evaluated from two aspects: external and internal. The external completeness relates to whether the data available to the surveillance system reflects the true number of cases affected by a given condition. One approach to evaluating external completeness consists of comparing at least two datasets from the different sources of information that are supposed to provide surveillance information on the same disease (e.g., laboratory and notification data for case reporting). The internal completeness refers to whether there are missing and/or unknown data fields in a surveillance database and can be defined as "the number of completed data fields out of the total number of data fields." A data extraction sheet was established based on the minimum data elements to be filled in the notification as recommended by WHO [16]. The data elements collected from the notification forms were age, gender, nationality, date of onset, date of diagnosis, date of receipt, address, travel history, travel destination, the collection of samples, the types of samples collected, and lab results. The data elements were analyzed based on their availability and legibility. An element was considered available if it was written in the form and considered legible if it was clear and readable. The principal investigators evaluated all the forms and a variable was judged legible if it was clear and readable by the researchers. To confirm the reproducibility of the grading, all the forms were double-checked across the researchers. Another surveillance attribute was the timeliness, which reflects the speed of transition between the different steps in a public health surveillance system.

Data analysis

The external completeness was calculated per month and the reporting facility counted the percentage of malaria notifications relative to the total number of malaria cases registered in laboratories over the country. It is worth mentioning that the surveillance team uses a supportive supervision approach to improve the quality of reporting where every laboratory is asked to send a record of all malaria cases on a monthly basis. We counted the number of notifications and the number of recorded cases. Then, we applied the formula: External completeness = Number of malaria notifications / Number of laboratory recorded cases * 100%.

Internal completeness was evaluated by calculating the proportions of complete data elements in the notification forms. The data elements of the notification forms were analyzed based on their availability and legibility. An element was considered available if it was written in the form and considered legible if it was clear and readable. The principal investigators evaluated all the forms and a variable was judged legible if it was clear and readable by the researchers. To confirm the reproducibility of the grading, all the forms were double-checked across the researchers.

**Figure 1:**
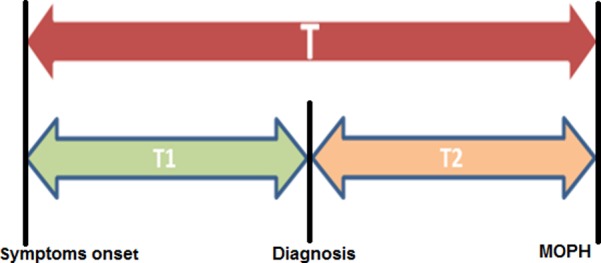
Time points of National Malaria Surveillance System in Qatar, 2016 T: timeliness between the onset of symptoms and the receipt of notification at the Ministry of Public Health T1: timeliness between the onset of symptoms and the diagnosis T2: timeliness between the diagnosis and the receipt of notification

The timeliness was evaluated by calculating the time lag between the malaria surveillance key time points: date of symptoms onset, date of diagnosis, and date of notification receipt by the central surveillance unit (Figure 1). Thus, three key time points were considered T (timeliness between the onset of symptoms and the receipt of notification at MoPH), T1 (timeliness between the onset of symptoms and diagnosis), and T2 (timeliness between reporting and the receipt of notification). A descriptive analysis of the surveillance attributes was performed using Microsoft Excel 2016.

## Results

**Figure 2:**
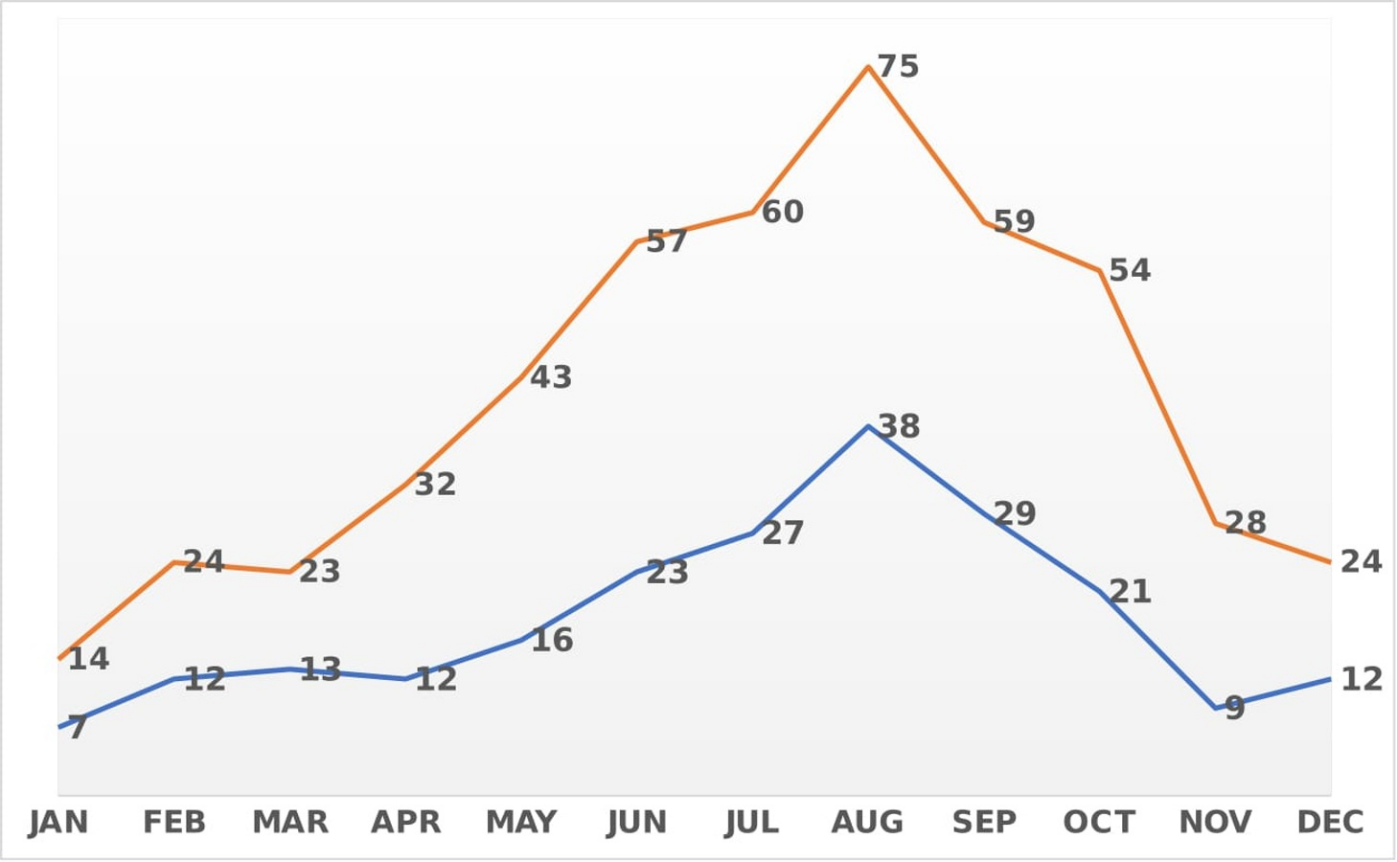
Distribution of laboratory-confirmed malaria cases and physician notifications by month in Qatar, 2016 Red: laboratory-confirmed cases Blue: physician notifications

Between January and December 2016, a total of 493 new malaria cases were notified through the laboratory-based reporting system. During the same interval, the central surveillance unit received 219 malaria notifications from physicians. The bulk of the notified malaria cases was registered in the months of July, August, and September while January showed the lowest number of notifications (Figure 2). Similarly, the frequency of diagnosed and reported cases shared a similar trend over time. Regarding the contribution of each institution to the overall notification process, the private sector ranked first and accounted for almost half of the notifications (47.5%). HMC was second in place with almost a quarter (24.6%) of the notifications arising from this party and followed by QRC that contributed with 16.1%. Additionally, PHCC (3.4%) and QP (0.4%) ranked last among the institutions involved in malaria notification.

The overall external completeness of malaria notifications from all reporting sites was calculated to be 219/493 (47%). Furthermore, in-depth analysis of each reporting health care facility revealed variable figures that ranged from a maximum of 93% for Qatar Red Crescent to a minimum score of 39% for HMC. However, within HMC, there was a diversity of scores. On the other hand, the private sector had an external completeness score of 83% and was followed by PHCC, which ranked third at 80 % (Table 1).

**Table 1:** External Completeness of National Malaria Surveillance System in Qatar, 2016 PHCC: Primary Health Care Corporation; HMC: Hamad Medical Corporation; AKH: Al Khor Hospital; AWH: Al Wakra Hospital; HGH: Hamad General Hospital

Health care facility	External completeness score
All health facilities	47% (219/493)
Qatar Red Crescent	93% (37/40)
Private sector	83% (109/132)
PHCC	80% (8/10)
HMC (overall)	39%
Cuban Hospital	83% (5/6)
AKH	42% (18/43)
AWH	22% (17/76)
HGH	9% (17/186)

In addition, ten data elements from the notification forms filled by physicians were analyzed for internal completeness and included age, gender, nationality, address, travel history, travel destination, diagnosis, sample collection, sample type, and lab results. The most frequently reported data fields were found to be age, gender, and nationality with a percentage of 99% or more. However, the least reported data components were found to be lab results, types of samples, sample collection, and travel destinations with percentages of 59%, 58%, 56%, and 41%, respectively. In addition, the internal completeness of the date of diagnosis and travel history valued at 80% and 83%, respectively. Regarding the contribution of each reporting facility to the overall internal completeness of the different data fields, QRC was the highest at 86.5% followed by the private sector (80.6%), PHCC (70%), HMC (67.2%), and QP (60%). In addition, the average internal completeness across the different data fields was about three quarters (76.2%).

Regarding the timeliness of Malaria surveillance system, the overall median time lag was six days for T, four days for T1, and two days for T2, with a broad range of zero to 127 days, two to 127 days, and one to 41 days, respectively. The timeliest notifications regarding the T key point were received from QP (four days) while QRC was responsible for the longest total time lag T (seven days). Regarding the T1 key point, the timeliest notifications arose from QP (three days) while the least timely notifications were received from PHCC (six days). Furthermore, the fastest contributors to the T2 key point were the private sector (one day) and QP (one day). On the other hand, PHCC (four days) was the slowest contributor to T2 (Table 2). Moreover, the overall median time of the aforementioned key points was three days for QP, four days for the private sector, six days for PHCC, 38 days for QRC, and 52 days for HMC.

**Table 2:** Timeliness of National Malaria Surveillance System in Qatar, 2016 *: T (median time lag between onset of Malaria symptoms and receipt of notification at MoPH) **: T1 (medican time lag between onset of symptoms and diagnosis of Malaria) ***: T2 (timeliness between diagnosis of Malaria and receipt of notification at MOPH) QP: Qatar Petroleum; QRC: Qatar Red Crescent; PHCC: Primary Health Care Corporation; HMC: Hamad Medical Corporation

Health care facility	Median time lag between Malaria surveillance key time points (days)			
	T*	T1**	T2***	
All health facilities	6	4	2	
QP	4	3	1	
QRC	7	4	2	
Private sector	5.5	4	1	
PHCC	6	6	4	
HMC	6	4	3.5	

## Discussion

The key findings of this evaluation study involved the completeness and timeliness of malaria notification forms as part of the national surveillance system in Qatar. The public sector, including HMC and PHCC, together contributed just over a quarter of the total notifications received by the surveillance section at MoPH. On the other hand, the public sector in Qatar is the major player in healthcare provision, where HMC provides about 75% of inpatient care through eight hospitals and PHCC provides provide 39% of outpatient care through 21 health centers [17].

In reference to the overall external completeness, the number of notified cases represents less than half of the total number of cases diagnosed at the laboratory level. We used a simple direct method to evaluate external completeness. Many studies applied other methods to evaluate the external completeness such as the capture-recapture method. A study conducted in Tunisia by Ben Alaya-Bouafif et al. revealed an external completeness level of 63.1% using the capture-recapture analysis [18]. Another study conducted in the Netherlands using the same method revealed a 56.4% external completeness level in 2003 up from 18.2% in 1998 as a result of the change in the infectious diseases law, mandating laboratory notifications of malaria in place of physicians; thus, significantly increasing the total number of malaria cases notified [19]. In this study, we could not use this method because data sources are dependent, which may underestimate the attribute.

Moreover, the data fields that were most lacking in the notification forms were laboratory results, type of sample collected, sample collection, and travel destination. This accounted for an overall 76.2% internal completeness of notification forms. On the other hand, a similar study that evaluated the communicable diseases’ surveillance system at Qatar’s Cuban Hospital found that the least completed data fields were address (68.1%) and place of work (60.5%) [20]. Even though the pregnancy status of female malaria patients is vital, it is not available as a data field in the national infectious disease notification forms of Qatar. This is probably due to the adaptation of the international data requirements to fit the local context, where the majority of malaria cases are imported through the influx of single male laborers from endemic countries [10]. However, the results of the mosquito and larvae survey studies mentioned earlier, along with the presence of imported malaria cases in the country, threaten to establish local transmission once more. Qatar’s expected hosting of the World Cup in 2022 further heightens the threat.

Regarding the timeliness of the surveillance system, the median time lag for T (time lag between the onset of Malaria symptoms and physician notification) was found to be six days, which was less than that recorded by the Cuban hospital study at (18.9) days [20]. Regarding the T2 time point, the median time lag between the reporting of a suspected case and the receipt of the notification by the surveillance section at MoPH was two days. The same figure was yielded by Akbari et al. during an evaluation study of the malaria surveillance system in Iran, in which the majority (90%) of cases were reported within two days to the second level (township center), noting that the timeliest notifications were those associated with a preset deadline [21]. Currently, the national surveillance system in Qatar is a paper-based process awaiting electronization. Adokiya et al. reported in their evaluation of the integrated disease surveillance and response system in northern Ghana that the timeliness of reporting at health facilities increased from 45% in 2012 up to 61% in 2013, marking the transition from paper-based reporting to an electronic system [22]. Additionally, an intervention reported by Quan et al. in South Africa focused on the mobile reporting of suspected malaria cases by trained nurses in rural area health clinics, whereby an SMS of the positive case would be sent to the local case investigator. Consequently, the information of about 78% of mobile-reported cases was processed into the information system within 24 hours, two to three weeks earlier than the standard reporting [23].

The longest median time lags regarding T2 were attributed to notifications arising from HMC (3.5 days) and PHCC (four days). The overwhelming patient load entertained at these two main governmental healthcare facilities may explain the aforementioned finding. Another possible explanation is related to the physicians’ knowledge and attitude toward the notification process. Similarly, a quasi-experimental study by Bawa et al. in Nigeria revealed that the training of health workers on the notifiable surveillance system led to increased completeness from 2.3% to 52% as well as timeliness from 0% to 42.9% [24].

Also, the median time lag between the onset of symptoms and diagnosis (T1) was four days, mainly due to delays from HMC (four days) and PHCC (six days). In addition to that, a study reported by Yoo et al. on the timeliness of the Korean notifiable diseases surveillance system showed that delayed reporting was mainly due to the time lag between disease onset and diagnosis. Thus, the authors proposed better public education on the notifiable disease of interest while also establishing clinical guidelines for physicians [25].

## Conclusions

Our study has identified several merits as well as areas of improvement in the National Malaria Surveillance System of Qatar, strengthening the importance of evaluation studies as strategic tools for operational development. The improvement in the completeness of notifications is required through periodic training of staff and monitoring of the quality of notifications. Likewise, the implementation of an electronic surveillance system can improve the overall quality of surveillance system with regards to malaria and other communicable diseases. Future evaluation studies should be more comprehensive and focus on other surveillance system attributes.
